# Life Insurance Companies and Physicians

**Published:** 1875-10

**Authors:** 


### Life Insurance Companies and Physicians.

We would call the attention of our readers to a note, published elsewhere, which presents in a concise manner in some very good advice in reference to the subject of the certificates of family physicians for Life Insurance Companies.

The officers of many of these companies seem to think that physicians have nothing to do but fill out certificates for them. Only a day or two since we were conversing with a prominent physician upon this subject. He informed us that he was almost daily in receipt of letters from companies asking as to the capabilities of some doctor whom they were about to appoint Examining Surgeon, and that it often took a large amount of valuable time to investigate and answer the letter as it should be. Still these companies never think of offering any fee, and some do not even condescend to send a stamp for the reply. Such requests should be consigned to the waste-paper basket.

